# Cohort Profile: the Cooperative Health Research in South Tyrol study

**DOI:** 10.1093/ije/dyaf064

**Published:** 2025-05-28

**Authors:** Rebecca Lundin, Roberto Melotti, Laura Barin, Martin Gögele, Stefano Lombardo, Antonio Fanolla, Paola Zuech, Johannes Rainer, David Emmert, Christian Fuchsberger, Deborah Mascalzoni, Alessandro De Grandi, Francisco S Domingues, Andrew A Hicks, Peter P Pramstaller, Cristian Pattaro

**Affiliations:** Eurac Research, Institute for Biomedicine, Bolzano, Italy; Eurac Research, Institute for Biomedicine, Bolzano, Italy; Eurac Research, Institute for Biomedicine, Bolzano, Italy; Centre for Medical Sciences, CISMed, University of Trento, Trento, Italy; Eurac Research, Institute for Biomedicine, Bolzano, Italy; Provincial Institute for Statistics of the Autonomous Province of Bolzano-South Tyrol (ASTAT), Bolzano, Italy; Observatory for Health—Autonomous Province of Bolzano-South Tyrol, Bolzano, Italy; Observatory for Health—Autonomous Province of Bolzano-South Tyrol, Bolzano, Italy; Eurac Research, Institute for Biomedicine, Bolzano, Italy; Eurac Research, Institute for Biomedicine, Bolzano, Italy; Eurac Research, Institute for Biomedicine, Bolzano, Italy; Eurac Research, Institute for Biomedicine, Bolzano, Italy; Center for Research Ethics and Bioethics, Uppsala University, Uppsala, Sweden; Eurac Research, Institute for Biomedicine, Bolzano, Italy; Eurac Research, Institute for Biomedicine, Bolzano, Italy; Eurac Research, Institute for Biomedicine, Bolzano, Italy; Eurac Research, Institute for Biomedicine, Bolzano, Italy; Eurac Research, Institute for Biomedicine, Bolzano, Italy

**Keywords:** population cohort, longitudinal, genetic, cardiovascular, metabolic, neurological, psychiatric, study representativeness

Key Features

- The Cooperative Health Research in South Tyrol (CHRIS) cohort was established to study genetic and lifestyle determinants of health and healthy aging in a single administrative district of the alpine Bolzano-South Tyrol province of Italy with a stable population, cooperative administration, shared single reference hospital, relatively homogenous customs and environment, and high healthy life expectancy.
- A baseline visit at the district reference hospital was completed by 13 393 adults aged ≥18 years (median 46, range 18–94, 54% female) in 2011–2018, providing socio-demographic, health, lifestyle, and exposure data from self-report questionnaires, interviews, and instrumental examinations, plus urine and blood samples for biobanking, DNA extraction, and molecular characterization (chip genotyping, exome sequencing, metabolomics, proteomics).
- To date, 4500 baseline participants have completed a visit as part of ongoing follow-up, providing most of the same data and samples as at baseline; additional data collection from wearable activity trackers and on prominent themes like COVID-19 have been introduced.
- Longitudinal data and biological samples have been collected in the CHRIS study district for over two decades, from 2003, encompassing sociodemographic information, medical history, clinical data, and molecular phenotypes.
- Dedicated requests for cohort data and samples for research purposes can be submitted via the CHRIS Portal (https://chrisportal.eurac.edu).

## Why was the cohort set up?

Italy’s autonomous South Tyrol province is uniquely adapted to longitudinal population studies of health and healthy aging with its alpine setting, self-governance, and control of most tax revenue, factors associated with improved health outcomes [1, 2], particularly in Europe. Italy is ranked 8th worldwide for life expectancy and 10th for healthy life expectancy at birth [3]. Among its regions, South Tyrol has the highest life expectancy at birth (83.7 years, 81.5 for males, 86.0 for females), and the autonomous province of Bolzano-South Tyrol has the highest healthy life expectancy at birth (69.3 years for males, 69.4 for females) [3]. South Tyrol has the second lowest probability of death from cancer, diabetes, and cardiovascular and respiratory diseases between the ages of 30 and 69 (7.2%) [4].

The South Tyrolean Val Venosta/Vinschgau administrative district (Vinschgau) has 13 municipalities with collaborative administration and a single reference hospital serving ∼36 000 inhabitants [5], a stable population with relatively homogeneous environment and lifestyles. This facilitates assessment of genetic health determinants [6], which, together with molecular and behavioral predictors, serve as ideal targets for studies of precision prevention, early detection, and treatment. Direct engagement with the local community strengthens participation in recall studies and fosters a proactive attitude to monitoring and promoting health.

The Cooperative Health Research In South Tyrol (CHRIS) population cohort study was established in Vinschgau to leverage these opportunities, investigating factors associated with common aging-related conditions, including cardiovascular, metabolic, and neuropsychiatric health in a dynamic, longitudinal manner. Results are relevant to rural European populations, given similar genetic backgrounds, lifestyles, and sociodemographic patterns, and representativeness can be enhanced by calibration methods.

## Who is in the cohort?

From 2011 to 2018, all Vinschgau adults 18 years and older able to attend an in-person study visit and complete informed consent were eligible for the CHRIS baseline. Invitation letters were mailed to all eligible inhabitants identified through publicly available electoral lists, and up to two reminders were mailed to non-responders. Further recruitment details are described elsewhere [7].

13 393 participants were recruited with a median age of 46 years (range: 18–94). 54% of participants were female, and 86% were born in the study area. 667 CHRIS baseline participants were previously enrolled in the Microisolates in South Tyrol (MICROS) study [8].

The cohort includes a high proportion of eligible individuals and is well-aligned with the reference population on socio-demographic, behavioral, and clinical aspects.

### Response rate estimates

Given census-like recruitment and stepwise inclusion of multiple municipalities at different times over 7 years of CHRIS baseline enrolment, we could not estimate response rate using standard formulas. We therefore developed overall and sex-stratified models with different assumptions regarding the target population, applying official figures for adults in Vinschgau provided by the Italian National Institute of Statistics (ISTAT) from 2011 to 2018.

A 42.6% response rate was estimated applying the narrowest eligibility assumptions, with higher participation among women than men (46.3% vs. 38.9%). Similar results were obtained from models with broader eligibility assumptions, with all response rate estimates exceeding the stated target of roughly 30% of the eligible population [7] (Supplementary Table S1).

### Reference population alignment

CHRIS participants were compared to the general Vinschgau adult population on socio-demographic characteristics, behaviors, and common disease prevalence.

**Figure 1.**
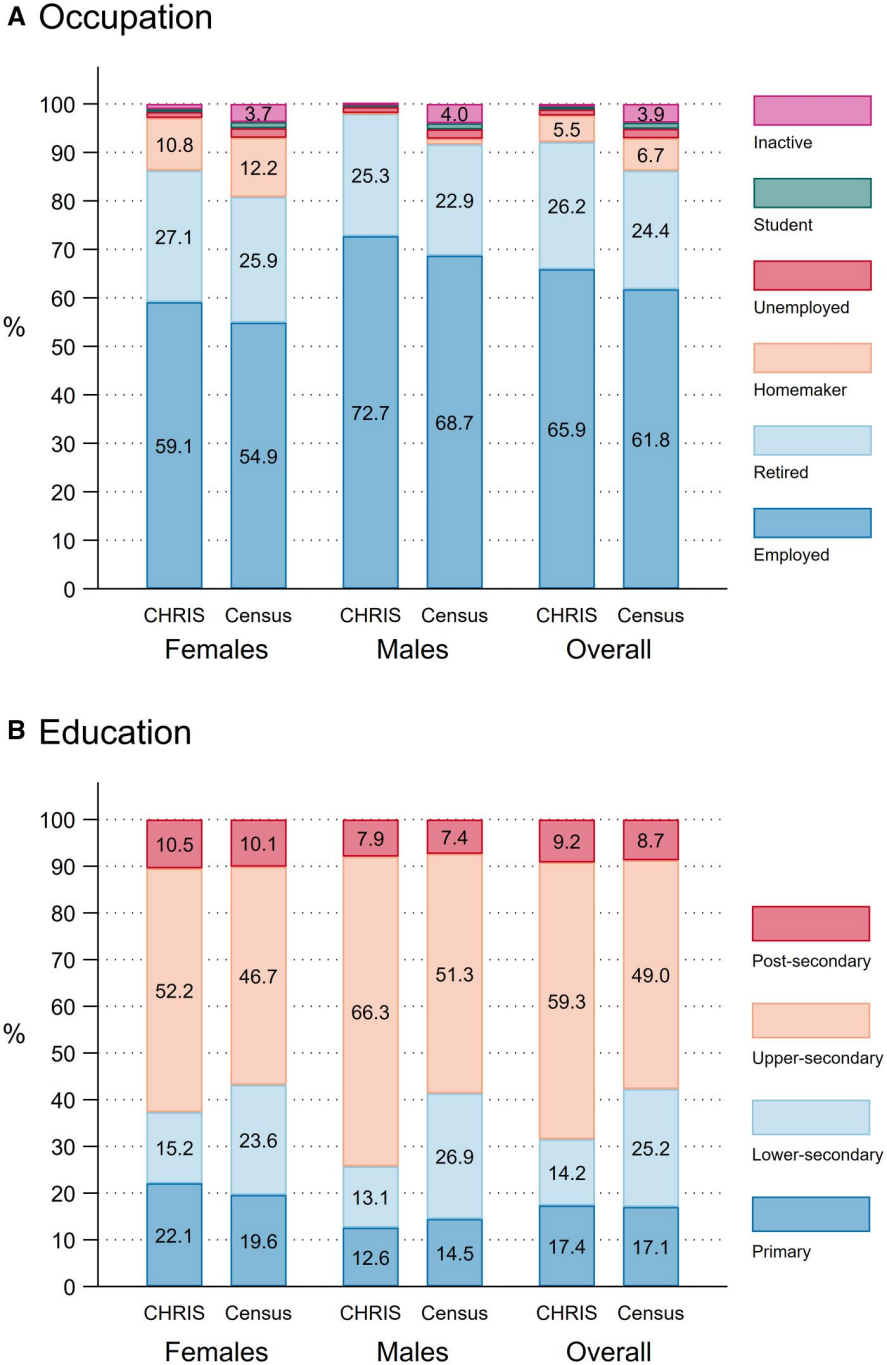
Representativeness of occupation and highest educational qualification in the Cooperative Health Research in South Tyrol (CHRIS) cohort, as compared to Vinschgau reference population. Bar labels display individual category proportion estimates. For readability, figures <2% are not displayed. (A) Occupational status. (B) Highest educational qualification. Reference population figures from 2018 population Census data. Cooperative Health Research in South Tyrol (CHRIS) figures calibrated by sex-age post-stratification weighting, using five age groups (25–34, 35–44, 45–54, 55–64, ≥65).

Occupation and educational qualification data from CHRIS were compared to 2018 ISTAT census data from Vinschgau adults 25 years and older, stratified by sex and age (Fig. 1, Supplementary Table S2a and b).

**Figure 2.**
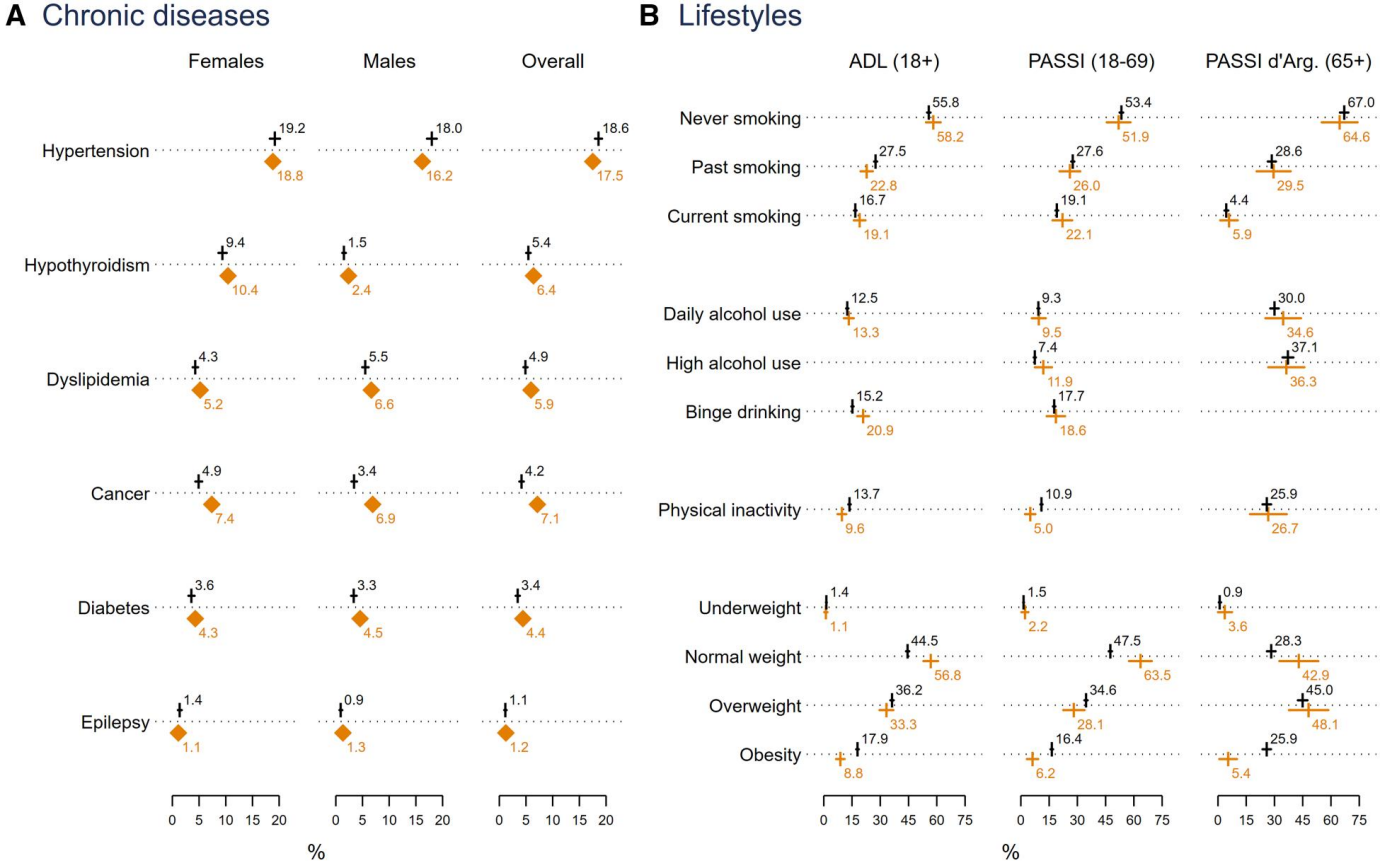
Representativeness of common chronic conditions and health-related lifestyle prevalence estimates in the Cooperative Health Research in South Tyrol (CHRIS) cohort as compared to administrative and survey data from the Vinschgau reference population. (A) Diamond markers represent population figures. Vertical bar markers with horizontal linear bars represent CHRIS estimates with 95% confidence intervals obtained by the Clopper–Pearson method. Reference population figures from 2016 Health Observatory administrative data. CHRIS figures calibrated by sex-age post-stratification weighting. (B) Vertical bar markers with horizontal linear bars located below the dashed horizontal line represent survey weight-adjusted figures with 95% confidence intervals provided by each data source, respectively. Vertical bar markers with horizontal linear bars located above the dashed horizontal line represent CHRIS estimates with 95% confidence intervals obtained by the Clopper–Pearson method. CHRIS figures calibrated by sex-age post-stratification weighting, according to the age bands of each comparable survey. ADL (aspects of daily life—administered by ISTAT) survey estimates obtained from survey participants 18 years and older in 2015 residing in rural South Tyrol, excluding five urban centers with more than 15 000 inhabitants. PASSI (Progressi nelle Aziende Sanitarie per la salute in Italia) survey estimates obtained from Vinschgau participants 18–69 years old, cumulatively responding to the surveys run between 2011 and 2018. PASSI d’Argento (PASSI in the elderly) survey estimates obtained from Vinschgau participants 65 years and older, cumulatively responding to the surveys run between 2016 (first year of the survey) and 2018.

CHRIS data on prevalence of six common diseases in CHRIS focus areas (cardiovascular, metabolic, and neuropsychiatric health) were compared with 2016 estimates based on administrative health data on Vinschgau adults reported by the South Tyrol Health Observatory (HO) (Fig. 2A, Supplementary Table S3a and b). CHRIS data in these analyses were self-reported except for hypertension, defined using drug data, and dyslipidemia, taken from blood values.

**Figure 3.**
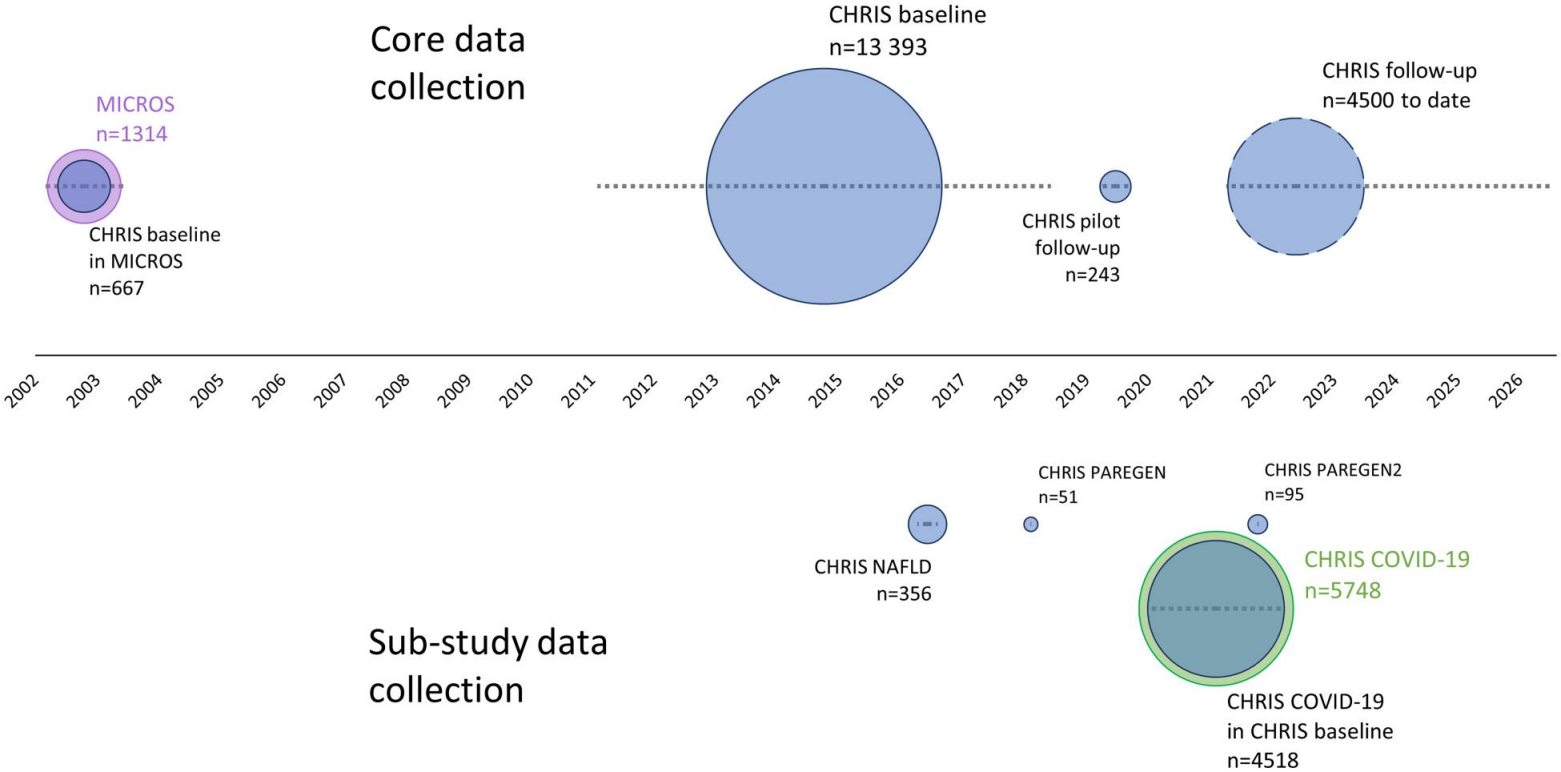
CHRIS cohort follow-up and sub-studies over time. Area of each circle corresponds to the number of participants in each data collection instance; where nested circles are used, internal circles represent CHRIS cohort participants while outer circles represent individuals participating in data collection but not the CHRIS cohort. Dashed lines indicate the period of data collection.

CHRIS data on lifestyles related to study focus areas were compared with 2015 data on South Tyrolean adults 18 years and older from the “Aspects of Daily Life” (ADL) survey administered by ISTAT, and data on Vinschgau adults 18–69 years old from 2011 to 2018 “PASSI” surveys and those 65 years and older from 2016 to 2018 “PASSI d’Argento” surveys administered by local health units and analyzed by HO (Fig. 3, Supplementary Table S4a and b). CHRIS data used in these analyses were self-reported, except BMI measured at baseline.

Fewer CHRIS participants reported being inactive, a student, or unemployed (0.6%, 0.6%, and 1.3%) than in the general population (3.9%, 1.3%, and 2.0%), while 65.9% and 61.8% reported being employed in CHRIS and in the general population, respectively.

More CHRIS participants reported higher secondary education (59.3%) than the general population (49.0%), while fewer reported lower secondary education (14.2% and 25.2%, respectively).

Disease prevalence estimates among CHRIS participants aligned with estimates based on administrative sources except for cancer, which was less frequent in CHRIS (4.2% vs. 7.1%).

Estimates of smoking and alcohol consumption were similar between surveys and CHRIS. Physical inactivity was reported by 10.9% of CHRIS participants below age 70 compared to 5.0% in PASSI. More CHRIS participants were classified as obese (17.9% vs. 8.8% ADL) and fewer as normal weight (44.5% vs. 56.8% ADL), possibly reflecting direct measurement compared to self-report.

## How often have they been followed up?

### Core cohort

CHRIS follow-up started in 2019 and stopped during the COVID-19 pandemic after recruiting 243 participants. Follow-up restarted in late 2021 and is anticipated to continue until 2027, with up to nine daily participants. Altogether, 4743 participants have completed a pilot follow-up or follow-up visit to date, with a 9.8-year median follow-up (range: 5.7–12.8). Among 3832 CHRIS baseline participants from 2011 to 2013, 67.1% participated in either pilot or core follow-up, with 3.9% deceased prior to invitation, 7.7% lost to follow-up, and 21.3% passive or active refusals.

### Prior study

The prior MICROS study enrolled 1314 residents of three Vinschgau municipalities 18 years or older in 2002–3 and 2007 [8].

### Substudies

Four substudies have been conducted in the CHRIS cohort (Supplementary Table S6).

The first was a “recall-by-phenotype” substudy on the relation between saliva and stool microbiota and Fibroscan-determined nonalcoholic fatty liver disease (CHRIS-NAFLD, *n* = 356) among 173 participants with type 2 diabetes mellitus and 183 sex-age matched participants without pre-diabetes or diabetes from late 2016 to early 2017 [9].

The second was a “recall-by-genotype” substudy to assess whether heterozygous mutation carriers of the Parkin gene, known to be involved in autosomal recessive Parkinson’s disease, had an increased risk of neurodegenerative symptoms. CHRIS-PAREGEN recruited 25 carriers and 26 noncarriers in 2018. A third substudy with the same aim, CHRIS-PAREGEN2, recruited 47 carriers and 48 noncarriers in 2022.

The fourth substudy was requested by the local community through their elected officials to understand the dynamics of the COVID-19 pandemic from 2020 to 2021 (CHRIS-COVID-19; 4518 CHRIS participants, 1230 cohabitants) [10–13].

CHRIS has been set up for further remote or passive phenotyping via online questionnaires, cell phone applications, or wearable technology, and 99.6% of participants have consented to recontact for further in-person data collection. These activities will be carried out depending on available funding.

## What has been measured?

From the beginning, CHRIS has placed special emphasis on molecular markers related to health and healthy aging, also collecting a wide range of sociodemographic, behavioral, and clinical information.

Measures conducted among all participants during the baseline CHRIS study center visit at the Schlanders/Silandro Hospital included fasting blood draw, urine collection, anthropometry, 20-min electrocardiogram, blood pressure, digital spiral drawing for tremor assessment, cognition, and olfaction assessment. Information on clinical history, health status, and exposures were collected via interviewer- and self-administered digital questionnaires, with some available in paper form prior to study visit. All data collection instruments are referenced elsewhere [7]. Laboratory analyses conducted on blood and urine samples included all main cardiovascular and metabolic biochemical parameters, antinuclear antibodies, and markers of iron metabolism, coagulation, and renal, thyroid and liver function (Table 1).

**Table 1.** Data collected, laboratory tests conducted, and biological material biobanked at CHRIS baseline and follow-up study visits

Domain	Assessment	Baseline	Follow-up
		*n* = 13 393	*n* = 4500
Sociodemographics and medical history	Marital status, education, occupation	● (I)	● (I)
	Socioeconomic status	–	● (I)
	Origin, familial relatedness (pedigree)	● (I)	–
	Birth/early-life exposures: birth weight, breastfeeding, congenital malformations	● (I)	–
	Gynaecology (for women): age at menarche, use of contraceptive pills, menopause status, use of hormone replacement therapy	● (I)	● (I)
	Medical history	● (I)	● (I)
	Medication (last 7 days)	● (I)	● (I)
	Parkinson’s disease symptom screening	● (I)	–
	Restless Legs Syndrome, screening and rating scale	● (Q)	–
	Jackson Heart Study, stroke symptoms form	● (I)^a^	● (I)
	Composite Autonomic Symptom Score	● (Q)^b^	● (Q)
	SF-12 Health Survey	–	● (Q)
	Cumulative Illness Rating Scale	–	● (Q)
	Early inflammatory arthritis detection tool	–	● (Q)
	German National Cohort infectious diseases screening	–	● (Q)
	COVID-19 infection and vaccination	–	● (I, Q)
Lifestyle	European Community Respiratory Health Survey II smoking module	● (I)^a^	● (I)
	Alcohol consumption	● (I)	● (I)
	Nutrition and coffee consumption	● (I)^c^	–
	GA^2^LEN study Food frequency Questionnaire	● (Q)^d^	● (Q)
	International Physical Activity Questionnaire -short version	● (Q)	● (Q)
Psychosocial aspects, Depression	Center of Epidemiologic Studies—Depression Scale	● (Q)	● (Q)
	State-Trait Anxiety Inventory (Form Y-2)	● (Q)	● (Q)
	MINI International Neuropsychiatric Interview: Depression, Mania	● (I)^e^	–
	Temperament Evaluation of Memphis, Pisa, Paris, and San Diego	● (Q)^f^	–
	Hypomania Checklist HCL-32 questionnaire for Energy, Activity and Mood	● (Q)^f^	–
	Life Orientation Test Revised	● (Q)^f^	–
	Perceived Stress Questionnaire	● (Q)^f^	–
	Childhood Trauma Screener	● (Q)^f^	–
Sleep	Pittsburgh Sleep Quality Index	● (Q)	–
	Munich Chronotype Questionnaire	● (Q)	–
	REM sleep behaviour disorder screening	● (Q)^g^	● (Q)
	UK Biobank sleep module	–	● (Q)
	Insomnia Severity Index	–	● (Q)
Pain	Pain Sensitivity Questionnaire	● (Q)	● (Q)
	Migraine symptoms	● (I)	–
	painDETECT (including extension to back pain)	–	● (Q)
	Mainz pain staging system	–	● (Q)
Anthropometry	Body weight, body height, bioelectrical impedance analysis	●	●
	Circumference measures	–	●
Cardiovascular examination	Blood pressure (office)	●^h^	●
	Blood pressure (20 min continuous, synchronized with electrocardiogram)	●^i^	–
	Electrocardiogram (10 s, 12 leads)	●	●
	Electrocardiogram (20 min, 12 leads)	●	–
Neurological examination	Digitalized spiralography	●^j^	–
	Algometer	●	●
	Sensory bedside Quantitative Sensory Testing (QST)	–	●
	Mini-Mental State Examination	●^b^	–
	Olfactory test (Sniffin’ sticks 16)	●^k^	●
Physical activity and fitness	7-day accelerometer	–	●
Laboratory analyses			
Electrolytes	Chloride, magnesium, potassium, sodium, calcium, phosphorus	●	●
Kidney function	Creatinine, uric acid	●	●
	Urea	–	●
Liver function	Alanine transaminase, aspartate transaminase, gamma-glutamyltransferase, alkaline phosphatase, albumin, bilirubin (total and direct)	●	●
Pancreas	Lipase	●	–
Lipid metabolism	Cholesterol, high density lipoprotein cholesterol, low density lipoprotein cholesterol, triglycerides	●	●
Glucose metabolism	Glucose, glycated hemoglobin (HbA1c)	●	●
Iron metabolism	Iron, ferritin, transferrin	●	●
Endocrine function/hormones	Cortisol	●	–
Thyroid function	Thyroid-stimulating hormone (TSH)	●	●
	Free triiodothyronine, free thyroxine (if TSH is out of reference range)	●^l^	●
	Anti-thyroid peroxidase antibodies	●	–
Inflammation	C-reactive protein high sensitive	●	●
Hematology	Complete blood cell count with differential	●	●
Coagulation	Prothrombin time, activated partial thromboplastin time, fibrinogen	●	●
	Antithrombin	●	–
Immunology	Antinuclear antibodies	●	–
	SARS-CoV-2 antibody	–	●
	Cytomegalovirus optical density, IgG	–	●
Cardiovascular health	Homocysteine	●^m^	–
Urine	Albumin, creatinine	●	●
	Urine physical and chemical examination	●	●
	Urine flow cytometry	●	●
	Urine clinical chemistry: sodium, potassium, chlorine	–	●
Biobank material	Serum, plasma, whole blood	●	●
	DNA	●	●
	Urine	●	●
	Saliva	●^n^	–

●, assessed in the whole cohort; (I), computer-assisted personal interview; (Q), self-administered questionnaire.

Available for ^a^11 691, ^b^8152, ^c^4640, ^d^8843, ^e^4688, ^f^4851, ^g^11 621, ^h^7137, ^i^6487, ^j^11 143, ^k^6964, ^l^598, ^m^11 310, ^n^1929 participants, respectively.

DNA was extracted from all consenting CHRIS participants, and genotyping was carried out in three batches using the Illumina OmniExpressExome chip (*n* = 5882), the Illumina Human Omni2.5Exome chip (*n* = 4887), and the Illumina-based OmniEURHD chip from Life & Brain GmbH, Bonn (*n* = 2694). Following quality control, the genotype dataset consists of 579 112 variants from autosomal chromosomes in 12835 samples. Whole-exome sequencing (WES) of 3840 CHRIS participants was performed using the xGen Exome Research Panel v1.0. After quality control, the WES dataset consists of 1 110 812 variants from autosomal chromosomes in 3597 samples.

From the CHRIS genotype dataset, imputed datasets were produced employing both the Haplotype Reference Consortium (HRC, https://ega-archive.org/datasets/EGAD00001002729) and TOPMed (https://imputation.biodatacatalyst.nhlbi.nih.gov/#) reference panels, yielding imputed datasets of 19 749 560 and 35 061 390 variants, respectively. An additional within-cohort imputed dataset was produced employing the CHRIS WES variants as reference panel [14], yielding an imputed dataset of 1 033 325 variants.

Large-scale metabolomics and proteomics data were acquired for a subset of CHRIS participants. Biocrates p180-based quantitative measurements of 175 serum metabolites and lipids and HILIC-based untargeted metabolomics data were generated for 6872 participants [15]. For 1055 participants, Biocrates MxP Quant 500-based concentrations for 650 metabolites and lipids were measured. A mass spectrometry-based approach quantified 148 highly abundant plasma proteins in 3632 participants [16, 17], and the Somalogic aptamer-based platform quantified 7301 proteins in serum samples from 4225 participants.

Residual aliquots of biological samples were biobanked for future use (Table 1). Molecular characterization of the baseline cohort is described in Table 2.

**Table 2.** Datasets derived from CHRIS baseline biological samples

Domain	Measurement	Baseline
Genomics	Illumina HumanOmniExpressExome chip, Illumina Human Omni2.5Exome chip, Illumina-based OmniEURHD chip	13 393
	High-coverage exome sequencing	3597
	mtDNA Copy Number quantification	9764
Microbiome	Saliva	1929
Metabolomics	Biocrates, targeted	7927
	Untargeted	6872
Proteomics	Scanning SWATH	3632
	SomaScan	4225
Other	Complement system activation	13 393
	Hepcidin	4783
	Renin angiotensin aldosterone system	2105
	Corticosteroids	3287

Familial relatedness information was collected in CHRIS baseline to construct a genealogy, including 8087 parent-offspring, 7018 full-sibling, 350 half-sibling, 1278 grandparent-grandchild, 16 105 avuncular (aunt/uncle-niece/nephew), and 29 746 first-degree cousin pairs.

Ongoing CHRIS follow-up collects mostly the same data as the CHRIS baseline. While some fixed traits are not re-measured (familial relatedness, birth/early life exposures), other measures have been eliminated to streamline study operations (20-min electrocardiogram plus continuous blood pressure, digital spiral recordings, selected survey instruments). Additions reflect current understanding and technical capacity, including wearable accelerometers to capture 1 week of physical activity and sleep data, quantitative sensory testing, and survey instruments on chronic pain, infectious diseases, inflammatory arthritis, and sleep. Self-report questions on COVID-19 vaccination, infection, and symptoms were added, and SARS-CoV-2 S and N antibodies were quantified. Most other laboratory parameters measured at baseline were included in follow-up (Table 1). Data and samples are collected during a Schlanders/Silandro Hospital study center visit, with some questionnaires available for prior completion as paper forms or secure individual links to online surveys.

In MICROS, interviewer-administered questionnaires collected family history, health history, and lifestyle exposures. Urine and blood samples were taken for routine biochemical analyses, genomic DNA isolation, and biobanking. Participants underwent clinical measures including anthropometry, blood pressure, and electrocardiogram (Supplementary Table S5).

Data and samples collected in the CHRIS pilot follow-up, CHRIS COVID-19, CHRIS-NAFLD, and PAREGEN substudies are described in Supplementary Table S6. All technical and organizational security measures foreseen by applicable legislation have been implemented to ensure privacy and personal data protection.

## What has it found?

CHRIS data and samples have been used to investigate associations between environmental, behavioral, and molecular factors and cardiovascular, metabolic, and neuropsychiatric outcomes, leveraging CHRIS’ breadth of phenotypes, deep molecular characterization, availability of longitudinal data, and ability to recall participants to expand data collection.

Associations were found between temperament and pain sensitivity [18], as well as nuanced relationships of intensity and duration of smoking with heart rate variability [19]. High total and specific animal protein intake were associated with lower kidney function [20]. Among the largest single-site targeted metabolomics datasets, CHRIS allowed discovery of the profound effect of hormonal contraceptives on the plasma proteome, indicating the need to account for this variable in proteomics studies of phenotypes associated with age or sex [17], as well as identification of metabolites significantly associated with age, sex, body mass index, diet and menopausal status [15].

CHRIS phenotype and comprehensive genotype data have been used to assess gene-trait associations, supporting previous findings and identifying novel genes and mechanisms for diseases and disease-related traits [14, 15, 21]. A GWAS assessing human host genomics and COVID-19 infection severity, combining data from 46 studies worldwide, including CHRIS, found 13 loci associated with more severe disease [13]. CHRIS also contributed to a GWAS meta-analysis among over 1 million individuals, resulting in a catalogue of genetic loci associated with kidney function [22], and to a GWAS of 5.4 million individuals from 281 studies providing a comprehensive map of specific genomic regions containing most variants associated with adult height [23]. A recent GWAS of functional activity of the three complement pathways using CHRIS data identified genes related to mouth ulcer risk and provides an extensive resource for investigating the role of complement in human health [24].

Longitudinal analysis of MICROS and CHRIS data allowed assessment of incident cardiovascular disease risk [25], while longitudinal data from the CHRIS COVID-19 substudy indicated the utility of symptom tracking for surveillance of novel pathogens in a population representative sample [12].

CHRIS data and samples have also contributed to methodological advances, including confirmation of pain sensitivity questionnaire internal validity and consistency between genders [26]. Machine learning clustering of renin-angiotensin-aldosterone system biomarkers from CHRIS was shown to identify specific antihypertensive treatments [27], and CHRIS data were used to develop programs for annotating untargeted metabolomics data [28] and to analyze familial aggregation of complex phenotypes in large pedigrees [29]. Dynamic consent, first introduced in CHRIS as a case study of feasibility and functionality in population-based cohorts, has provided a valuable and flexible model for consent nationally and internationally [30].

## What are the main strengths and weaknesses?

A key strength of CHRIS is in-depth, accurate measurement of a wide range of cardiovascular, metabolic, and neuropsychiatric phenotypes through repeated in-person study visits combined with genotype and multi-omics data. The rural alpine setting with high healthy life expectancy is uniquely adapted to the study of determinants of health and healthy aging, while a relatively stable population with homogenous environments and lifestyles facilitates identification of genetic determinants.

The strong ties researchers have developed with the local health system and particularly the local community facilitate multiple recall studies with high participation, ranging from 33.7% for CHRIS COVID-19 to 86.2% for CHRIS PAREGEN, and allowed the design and implementation of CHRIS COVID-19 upon community request. With no medical campus in the province, CHRIS also provides opportunities for biomedical education, bringing together clinicians for training and networking, and developing graduate and post-graduate programs.

Weaknesses of the CHRIS study include potential bias due to lengthy baseline and follow-up recruitment periods, necessitated by in-depth data collection and logistical constraints. We found that inclusion of day of participation as a random effect can avoid overestimation in heritability analysis [31]. As with all population-based cohort studies, selection bias is also a concern, though our assessment of the representativeness of the CHRIS cohort across several sociodemographic characteristics, chronic diseases, and lifestyles indicates that calibrated weights can be used to produce reliable population-level estimates. Finally, while much larger population-based cohorts have been established, our publications show that CHRIS is sufficiently powered for many investigations and is able to provide important contributions to pooled analyses.

## Can I get hold of the data? Where can I find out more?

Data and samples can be requested for clearly defined research via the CHRIS Portal (https://chrisportal.eurac.edu). For questions, contact rebecca.lundin@eurac.edu.

## Ethics approval

The Ethics Committee of the Healthcare System of the Autonomous Province of Bolzano-South Tyrol approved the CHRIS baseline protocol on 19 April 2011 (21-2011), the CHRIS follow-up protocol on 13 June 2019 (56-2019) with updates approved on 16 October 2019, 13 October 2021, and 19 July 2023, the CHRIS-NAFLD protocol on 22 September 2016 (85-2016), the CHRIS PAREGEN protocol on 19 July 2018 (56-2018), the CHRIS COVID-19 protocol on 27 May 2020 (53-2020) with an approved update on 22 July 2020, and the CHRIS PAREGEN 2 protocol on 19 May 2021 (66-2021) with updates approved on 17 November 2021 and 24 August 2022.

The Provincial Ethics Committee of Bolzano-South Tyrol approved the MICROS protocol on 11 November 2003 (23.5 DrMVH/31.05.07.14/19644) with an approved update approved by the Ethics Committee of the Healthcare System of the Autonomous Province of Bolzano-South Tyrol 18 September 2013.

The study conforms to the Declaration of Helsinki and to national and institutional legal and ethical requirements.

## Supplementary Material

dyaf064_Supplementary_Data

## Data Availability

Data can be shared upon request.
